# Recombinant human B cell repertoires enable screening for rare, specific, and natively paired antibodies

**DOI:** 10.1038/s42003-017-0006-2

**Published:** 2018-01-22

**Authors:** Saravanan Rajan, Michael R. Kierny, Andrew Mercer, Jincheng Wu, Andrey Tovchigrechko, Herren Wu, William F. Dall′Acqua, Xiaodong Xiao, Partha S. Chowdhury

**Affiliations:** 1 Antibody Discovery and Protein Engineering, MedImmune, Gaithersburg, MD 20878 USA; 2 Research Bioinformatics, MedImmune, Gaithersburg, MD 20878 USA; 3 Present Address: RegenxBio Inc, Rockville, MD 20850 USA; 4 Present Address: Bristol-Myers Squibb, Redwood City, CA 94063 USA; 5 Present Address: Sanofi-Genzyme R&D Center, Framingham, MA 01701 USA

## Abstract

The human antibody repertoire is increasingly being recognized as a valuable source of therapeutic grade antibodies. However, methods for mining primary antibody-expressing B cells are limited in their ability to rapidly isolate rare and antigen-specific binders. Here we show the encapsulation of two million primary B cells into picoliter-sized droplets, where their cognate V genes are fused in-frame to form a library of scFv cassettes. We used this approach to construct natively paired phage-display libraries from healthy donors and drove selection towards cross-reactive antibodies targeting influenza hemagglutinin. Within 4 weeks we progressed from B cell isolation to a panel of unique monoclonal antibodies, including seven that displayed broad reactivity to different clinically relevant influenza hemagglutinin subtypes. Most isolated antibody sequences were not detected by next-generation sequencing of the paired repertoire, illustrating how this method can isolate extremely rare leads not likely found by existing technologies.

## Abstract (web summary)

Saravanan Rajan et al. describe a high-throughput method for isolating unique human monoclonal antibodies using picoliter sized droplets containing primary B cells. They show this approach can rapidly drive selection towards novel antibodies against clinically-relevant influenza hemagglutinin subtypes.

## Introduction

Antibodies are among the fastest growing therapeutic classes within the biopharmaceutical industry^1^. Whereas most approved therapeutic antibodies have been obtained by engineering rodent antibodies, highly potent antibodies have recently been identified within humans against many diseases, including microbial infection^2–5^, autoimmunity^6–8^, and cancer^9^. Because these antibodies are elicited in human responses to disease, they are believed to be safer, less immunogenic and in general more translatable to human therapy^10^. However, the B cells producing these therapeutic antibodies tend to be rare in convalescent patients, making their discovery very challenging. Adding to this challenge is the fact that antibodies are heterodimeric proteins whose specificities are encoded by unique pairs of heavy-chain and light-chain transcripts. Technologies that preserve this native pairing are therefore best suited to recapitulate the functional characteristics of naturally produced antibodies. These methods generally fall into two categories. First, B cells can be cultured in individual wells and their conditioned media screened for function, or their antibody genes directly cloned. However, maintaining large numbers of monoclonal cultures for extended periods of time is laborious, expensive and limits the screening to a fraction of the B cell repertoire^11^. A more recent development uses next-generation sequencing (NGS) to profile the paired repertoire from millions of B cells^12,13^. Though the number of cells that can be sequenced with this method is high, inferring antigen-specificity from sequencing information is very challenging, especially from humans who are constantly exposed to a large diversity of antigens. Moreover, validating leads requires gene synthesis, cloning, and expression which can create a severe bottleneck in the number of candidates that can be functionally assessed^14^. Both of these methods therefore suffer from low screening throughput that overwhelmingly under-samples the ~10^7^ B cells obtained from a typical blood draw. There is an urgent need for a discovery engine that adequately mines the natural B cell diversity to rapidly isolate antigen-specific antibodies from human patients.

**Fig. 1:**
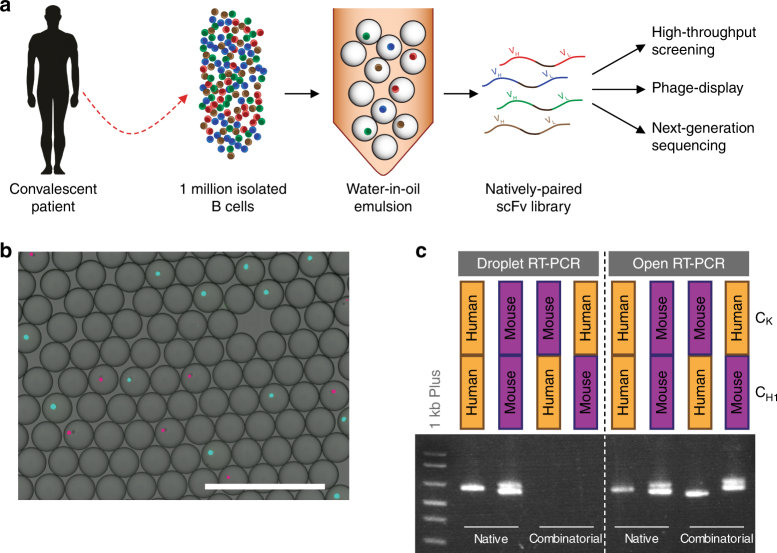
Strategy for generating natively paired libraries. **a** Isolated B cells are purified from convalescent donors and encapsulated into water-in-oil droplets with RT-PCR reagents such that cognate V_H_ and V_L_ domains are amplified and linked. The resulting amplicon forms an expression-ready scFv which can be directly expressed for screening, displayed on phage for selections, and deep sequenced for repertoire characterization. **b** Single-cell encapsulation using droplet microfluidics. Cells were stained with CellTracker Red and Green dyes (pseudocolored magenta and cyan, respectively), mixed, and encapsulated into droplets at a density that favored single-cell encapsulation. Each droplet forms an independent reaction vessel in which that cell’s cognate V genes can be amplified and paired. Scale bar = 400 μm. **c** Validation of native chain pairing during droplet RT-PCR. Primary human and mouse B cells were mixed and their C_H1_–C_K_ domains linked in either encapsulated (“droplet”) or pooled (“open”) RT-PCR. The resulting amplicons were purified and chain pairing was determined using specific nested primer combinations. Correctly paired species was only obtained using encapsulation, whereas the scambled format generated all possibile combinations in relatively equal amounts

Here we report the creation of a microfluidic platform that pairs cognate V_H_ and V_L_ transcripts from millions of single cells into “expression-ready” scFv libraries, while still maintaining the ability to profile the paired repertoire by NGS (Fig. 1a). We coupled these recombinant repertoires with the enormous screening power of phage-display to rapidly enrich for antigen-specific clones. Using this method, we interrogated the antibody repertoires from healthy individuals by screening for influenza hemagglutinin (HA) binding and selected for a panel of cross-reactive leads targeting multiple HA subtypes.

## Results

### Cognate chain pairing from encapsulated primary B cells

Our approach involves encapsulating single B cells into water-in-oil droplets of ~400 pl in volume (Fig. 1a). We used glass microfluidic chips with pressure pumps to reliably generate evenly sized droplets at high rates, such that one million B cells could routinely be encapsulated within 40 min. The architecture of the microfluidic chip is designed to merge two streams of aqueous fluids: one carrying a suspension of B cells and the other containing reagents for one-step reverse transcription and overlap-extension PCR. We titrated the B cell suspension to achieve ~1 cell for every 10 droplets which, based on Poisson statistics, will result in single-cell encapsulation with >95% probability (Fig. 1b). As the contents of droplets cannot easily be modified once they have formed, we optimized a one-pot reaction mixture to perform cell lysis, reverse transcription, PCR amplification of V_H_ and V_L_ domains, and their linking by overlap-extension PCR. Though it has been reported by several groups that cell-based RT-PCR is not feasible in volumes of <5 nl^12,13,15,16^, we have successfully amplified Ig transcripts directly from B cells in picoliter-sized droplets. Achieving this required extensive optimizations of the reaction components and conditions. For instance, out of 11 commercially available One-Step RT-PCR reaction mixes tested, only one reliably generated stable droplets and reproducibly yielded RT-PCR amplicons of human GAPDH (Supplementary Table 1). We confirmed that cells were robustly lysed upon addition of RT-PCR buffer and incubation at 50 °C by Trypan Blue staining (Supplementary Fig. 1), release of cytosolic dyes and detection of nuclear material by SYBR-Green (Supplementary Fig. 2).

To validate our approach at achieving single-cell encapsulation and cognate chain pairing, we first used a mixture of primary human and mouse B cells and designed primer sets to amplify and link the C_H1_ and C_κ_ domains (Fig. 1c; Supplementary Fig. 3, Supplementary Table 2). We added complementary overhangs to the reverse-C_H1_ and forward-C_κ_ primer sets (the “inside” primers) to fuse the two domains with a (Gly_4_-Ser)_3_ linker used in our previously described scFv libraries^17^. Using this design, we mixed equal amounts of primary mouse and human B cells and generated single-chain amplicons in either encapsulated or un-encapsulated formats. The four possible products—native (hC_H1_–hC_κ_ and mC_H1_–mC_κ_) and combinatorial (hC_H1_–mC_κ_ and mC_H1_–hC_κ_)—were easily discernible by nested PCR using species-specific primers and were confirmed by Sanger sequencing. As expected, the combinatorial format produced all possible products but, strikingly, only natively paired amplicons were generated with encapsulation (Fig. 1c; Supplementary Fig. 3).

### Natively paired antibody libraries from human B cells

**Fig. 2:**
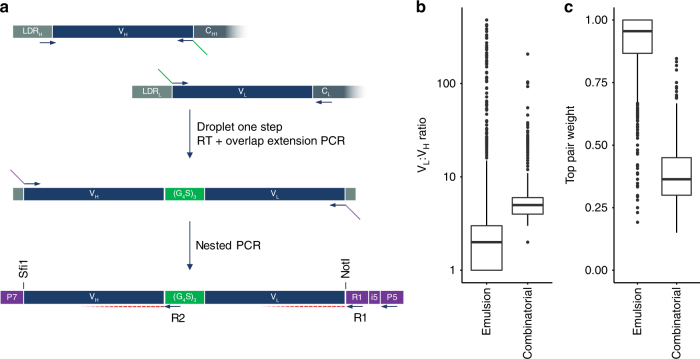
Generating natively paired libraries for screening and next-generation sequencing (NGS). **a** V_H_ and V_L_ domains from each encapsulated B cell are amplified with specific primer sets and paired in-frame via complementary overhangs (green). A nested PCR with V_H_ FR1 and V_L_ FR4 primers generates full-length scFv with overhangs (purple) to enable barcoded paired MiSeq sequencing. V_H_ and V_L_ FR3, CDR3 and FR4 are sequenced with R2 and R1 primers, respectively, while the standard P5-specific primer provides the index read to enable demultiplexing. The amplicons can be cleaved of adapter sequences via Sfi1/Not1 restriction sites for subcloning into expression or phagemid vectors for library generation. **b**, **c** NGS data from a representative dataset where scFv libraries from one million stimulated B cells were generated in either emulsion or combinatorial fashion. **b** Emulsion libraries favored a 1:1 V_L_:V_H_ ratio whereas combinatorial libraries were scrambled. **c** In the case where multiple pairings were seen, the dominant partner accounted for 96% of sequences in the emulsion library, whereas partners were more evenly distributed in the combinatorial library

Having confirmed that we could successfully pair cognate chains from single primary B cells, we proceeded to capture the paired immunoglobulin repertoire into an expressible format. Primer sets for multiplex amplification of all known human V and J genes were computationally designed from IMGT consensus sequences. We designed 92 primers to amplify the 542 functional human V and J alleles with the appropriate overhangs for scFv generation (Supplementary Data 1). One million total B cells isolated from the blood of a healthy donor were mixed with 10,000 IM-9 cells (1%) before being encapsulated with our optimized RT-PCR mix to generate a natively paired amplicon library, consisting of (from 5′ to 3′) part of the V_H_ leader sequence, V_H_, (Gly_4_-Ser)_3_ linker, V_L_, N-terminus of C_L_. This product was then used as template for nested PCR with V_H_ FR1 and V_L_ FR4-specific primer sets to generate a full-length scFv amplicon library (Fig. 2a). As a second validation of correct chain pairing, we used a forward primer specific to the IM-9 CDRH3 sequence (RRGVTDIDPFDI; IM-9-CDRH3-Fwd) with a generic reverse primer (R1; Supplementary Table 3) to amplify all V_L_ sequences that paired with the IM-9 heavy chain. The resulting amplicon was cloned and analyzed by Sanger sequencing, which showed correct pairing with the known IM-9 V_L_ (QHYNRPWT) in 97/101 colonies (96% pairing accuracy), illustrating that even with a 100-fold abundance of competing B cells, our method is able to maintain correct chain pairing.

We then set out to use this method in a discovery campaign to isolate antibodies against influenza hemagglutinin, an antigen to which humans are commonly exposed. Two million B cells were separately isolated from the blood of two healthy donors: total B cells from Donor 1 and switched memory B cells from Donor 2. For each donor, one million cells were encapsulated as above to generate natively paired emulsion libraries. The remaining million cells were used to build combinatorial scFv libraries by separately amplifying V_H_ and V_L_ domains from purified RNA and pairing them by standard overlap-extension PCR, using the same primer set.

To obtain an in-depth assessment of the captured repertoire, our nested PCR primers contain barcoded overhangs that enable NGS on the Illumina MiSeq (Fig. 2a). We designed a custom priming strategy to obtain paired 300 bp reads of the 3′ ends of V_H_ and V_L_, which generated high-quality sequences in regions mapping to FR4, CDR3, and FR3 (Supplementary Fig. 4). Whereas the V_L_ read was obtained using a priming site introduced at the 3′ end of the construct, the V_H_ read required an internal primer annealing to the (G_4_S)_3_ linker sequence (Supplementary Table 3). Sequencing reads were quality filtered and translated before clustering sequences that were greater than 88% identical, which falls within the range of reported thresholds used to correct for sequencing errors while minimizing the loss of truly unique sequences (reviewed in ref.^18^).

Sequences were annotated using IgBLAST^19^ to assign germline families and delineate CDR3 regions. We recovered a total of 212,018 and 2,549,415 unique CDRH3:CDRL3 clusters for the two emulsion and combinatorial libraries, respectively (Supplementary Table 4). We observed similar distributions of CDRH3 and CDRL3 lengths (between 3–21 and 8–11 amino acids, respectively) between the emulsion and combinatorial libraries (Supplementary Fig. 5a, b and Supplementary Fig. 6a, b). The emulsion libraries also included most known V and J germline families (Supplementary Fig. 5c, d, e and Supplementary Fig. 6c, d).

As a third validation that our system preserves chain pairing, we determined the number of unique CDRL3 sequences that were paired with each CDRH3 sequence. As expected, the combinatorial libraries displayed promiscuous pairing, with each CDRH3 sequence paired with a median of 5–9 unique CDRL3 sequences (Fig. 2b; Supplementary Table 4). Given that the sequencing depth (10^6^) vastly under-samples the theoretical sequence diversity of the combinatorial libraries (10^12^), the true rate of combinatorial pairing would likely be considerably higher. This was in stark contrast to the emulsion libraries, where we observed a median of 1:2 CDRH3/CDRL3 pairing with narrow distribution. In cases where multiple pairings were detected, top-pair analysis determined a 96% accuracy in V_H_–V_L_ pairing (Fig. 2c). Top-pair analysis has been used to validate cognate chain pairing in a previously published method that generates amplicons only suitable for sequencing, but not for screening^13^. Interestingly, using similar sequencing depth and starting cell numbers we have found the pairing efficiency to be significantly better in our approach (*p* < 0.001; Supplementary Fig. 7, Supplementary Table 5). This could be because our method covalently links V_H_ and V_L_ domains within the droplets, rather than using an intermediate bead binding step where exchange of mRNA species between beads might occur^20^.

### Selection for cross-reactive anti-hemagglutinin antibodies

**Fig. 3:**
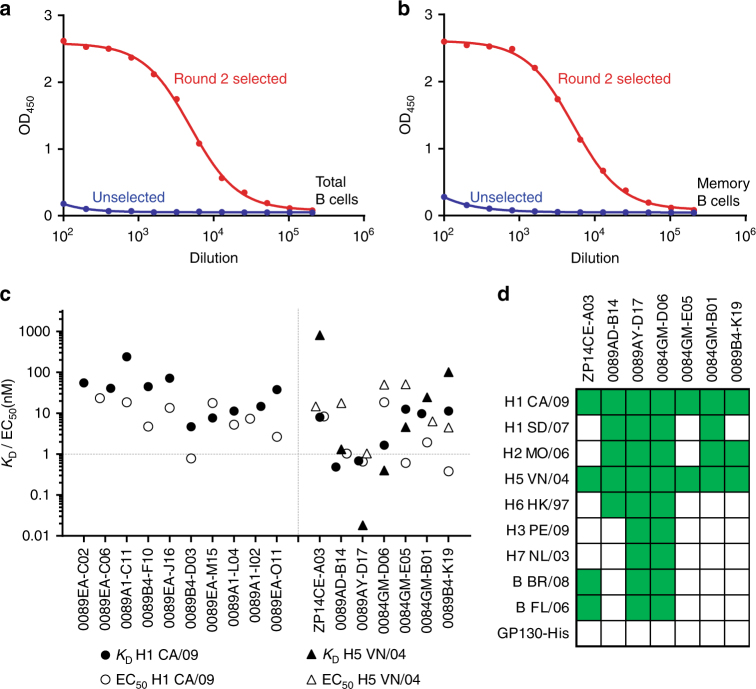
Phage-display enrichment for antigen-specific antibodies. Emulsion libraries obtained from total (**a**) and memory (**b**) B cells were subjected to two rounds of phage-display panning on hemagglutinin H1 (A/California/07/2009 H1N1) and enrichment was measured by polyclonal phage ELISA, where the unselected library is shown in blue and the enriched library in red. **c** Specific binding data for 17 monoclonal scFv-Fc antibodies against two different influenza hemagglutinin subtypes. Monovalent affinities (measured by biolayer inferometry) and bivalent EC_50_ binding (measured by ELISA) are shown as filled and open symbols, respectively, against H1 (A/California/07/2009 H1N1—circles) and H5 (A/Vietnam/1203/2004 H5N1—triangles). **d** Binding data of cross-reactive antibodies against a panel of hemagglutinin antigens from Group 1 and 2 subtypes of influenza A and both lineages of influenza B, where observed binding by biolayer inferometry is shown in green (GP130-his was included as a negative control). Full virus strain designations specified in Methods

The emulsion and combinatorial libraries were bulk subcloned into a phagemid vector^17^ to construct phage-display libraries of approximately 10^8^ transformants. Monoclonal phage ELISA against the myc tag fused to the scFv indicated that most transformants in the libraries displayed scFv well, with positive display seen for 90–99% of clones (Supplementary Table 6). The four libraries were subjected to two rounds of enrichment on the 2009 pandemic influenza A hemagglutinin (A/California/07/2009 H1N1). Polyclonal phage ELISA confirmed robust enrichment for specific binders regardless of the B cell source (Fig. 3a, b; Supplementary Fig. 8). Of note, while the combinatorial libraries showed an overall stronger specific enrichment, NGS of the enriched libraries revealed a strong bias (85%) for the IGHV1-69 germline family as compared to the corresponding emulsion library (15%; Supplementary Fig. 9). It has previously been shown that IGHV1-69 containing antibodies can contact group 1 hemagglutinin subtypes through heavy-chain interactions alone^2^. It is therefore likely that the enrichment of combinatorial libraries was driven by selecting for V_L_ partners to IGHV1-69 that expressed or folded well in bacteria and highlights a key bias with combinatorial libraries.

To specifically enrich for cross-reactive antibodies, we panned the first round output from the emulsion libraries on a non-circulating group 1 subtype, influenza A hemagglutinin H5 (A/Vietnam/1203/2004 H5N1). The enriched scFv pools were bulk subcloned into an scFv-Fc expression vector^21^ and screened by ELISA. Of the 5,632 clones screened, we identified 320 clones that show specific binding to H1, consisting of 17 unique antibodies that represent different germlines and display a range of affinities, with several in the picomolar range (Fig. 3c; Supplementary Data 2). This includes seven unique antibodies that bind to both antigens used during panning, most of which display an even greater breadth of cross-reactivity while showing no binding to a negative control antigen, GP130-his (Fig. 3d). It is interesting that two of these antibodies (0089AY-D17 and 0084GM-D06) display specific binding to all 10 hemagglutinin subtypes we tested, including subtypes from influenza A (Group 1 and 2) and both lineages of influenza B (Fig. 3d). Competition studies using biolayer inferometry revealed that these antibodies do not compete for binding with the published cross-reactive antibodies FI6v3^22^, MEDI8852^23^, 1N23^24^, and CR9114^25^, suggesting that they may define novel cross-reactive epitopes (Supplementary Fig. 10). Finding such universal anti-influenza antibodies was unexpected, particularly within healthy donor samples, yet underscores the power of our method to isolate rare specificities through deep mining of the repertoire. It is possible that we selected for an epitope unique to the recombinant HA, such as the Foldon-His domain added to enable trimerization and facilitate purification. To test this, we expressed a different protein (RSV-F) fused to the identical Foldon-His domain and did not observe binding to our antibodies (Supplementary Fig. 11). It will be interesting to see the results from structural studies that fully characterize the identity of this epitope.

To test for neutralization activity, we expressed and purified the seven cross-reactive antibodies as IgG1 heterodimers. Two of these (ZP14CE-A03 and 0084GM-D06) did not retain binding after conversion and were excluded from further analysis. This falls within the expected attrition rate we have seen when converting between scFv-Fc and IgG1 formats^26^. For the remaining five antibodies, we performed microneutralization assays using the seasonal H1N1 A/California/07/2009 virus or the non-seasonal H5N1 A/Vietnam/1203/2004 virus isolated from human infection. Our antibodies did not exhibit neutralizing activity against either virus at the highest concentration tested (1 mg ml^−1^; Supplementary Fig. 12), though we cannot rule out the possibility of these antibodies acting via indirect antiviral mechanisms such as Fc-mediated effector function. Although our goal was to drive discovery of cross-reactive antibodies through selection of natively paired libraries, using this technique with functionally profiled B cell sources (for instance using AMBRA^27^) could improve the chances of also isolating antibodies with specific mechanistic properties.

To ascertain the relative frequency of our leads within the captured B cell repertoire, we searched for their respective CDRH3:CDRL3 pairs within our sequencing dataset, allowing for up to four amino acid mismatches to account for possible PCR- and sequencing-induced mutations. A recent addition to the field of repertoire sequencing has been the introduction of universal molecular indices^28^ to correct for such mutations and ideally our strategy would have included this approach. However, this was a challenge since terminally appended barcodes would be removed during nested PCR, and incorporating internal barcodes would likely compromise the functional integrity of our scFv library. Even with this permissive cutoff, only one of the 17 antigen-specific sequences was observed among the 212,018 unique paired sequence clusters, implying that the remaining hits were too rare to be detected by NGS. This sequence (0089EA-C02) accounted for 2 out of 4,820,834 mapped V_H_–V_L_ reads (Supplementary Table 4). Following selection, we found this clone repeated in 32 out of the 5,632 clones screened, an enrichment of approximately 14,000 fold. It is possible that more abundant antigen-specific sequences existed within the repertoire, but were not selected because of differences in expression and folding of human antibodies in bacteria^29,30^. This could be readily addressed in future iterations of the technology by employing other platforms for displaying native human antibodies, such as yeast-display^31^. As this platform depends on successful PCR from gene-specific primers, it should also be noted that antibody genes mutated within the primer binding sites may be excluded from the resulting library, though it is possible that ancestral antibodies of equal activity yet having fewer mutations^23,32^ could still be captured. Nevertheless, this particular set of leads could not have been predicted from sequencing information alone. If one assumes that the scarcity of these leads determined by NGS represents that within the original B cell pool, it would also seem very unlikely that these leads could be found through standard methods of culturing and screening individual B cells.

## Discussion

We have developed a technology that can rapidly capture the native repertoire from millions of primary human B cells into a powerful and sensitive screening platform, with significant implications for therapeutic antibody development, immune repertoire characterization and can have wide application in rational vaccine design. By linking the variable domains into a translatable scFv format we can now combine the strengths of multiple technologies: using the immense screening power of display platforms to mine the full richness of a naturally evolved antibody response. The process outlined here is one of the fastest for antibody discovery from natural repertoires. A single researcher can rapidly progress from millions of primary B cells to specific monoclonal antibodies within 4 weeks. This could be especially valuable for combating emerging infectious diseases, such as the recent Ebola and Zika outbreaks. The method is also very robust, as to date we have successfully recovered scFv libraries from over 20 separate encapsulations of one million B cells from various donors (a subset of which is shown in Supplementary Fig. 13). These libraries constitute a renewable resource that can be expanded as new donors are added, panned repeatedly against a multitude of targets (including whole bacteria or tumor tissue), or archived indefinitely for future use. Large-scale efforts that use NGS to predict antibody function could particularly benefit, such as the recently launched Human Immunome Program^33^. This project aims to sequence the expressed antibody repertoires from 1000 individuals and infer vaccine reactivity based on sequencing information alone. An exciting addition to this project could be to use the method outlined here to build display libraries from these individuals, such that one could directly measure the reactivity of the human repertoire to any number of vaccine candidates.

While the steps outlined here enable isolation of antibodies from human B cells, it can readily be extended to isolate antibodies from any species for which V-gene sequence information is available. This can be particularly useful for expanding the breadth and depth of the hybridoma technology, where low fusion efficiencies (<0.5% ^34^) lead to significant loss of repertoire. The technology can also be applied for generating monoclonal antibodies from organisms for which myeloma fusion partners are not available. T cell receptor (TCR) repertoires (consisting of paired α/β or δ/γ chains) could also be captured and screened in a similar recombinant format, as single-chain TCR has been shown to be amenable to selection by phage-display and yeast-display^35,36^. Finally, the method presented here uses equipment and reagents that can be purchased from commercial vendors at prices well within the reach of most laboratories. This opens the door for widespread mining and archiving of antibody repertoires for *de novo* identification of functional antigen-specific antibodies from natural sources.

## Methods

### Primer design

All primers were designed to anneal with a minimal melting temperature of 60 °C and, where possible, were consolidated to have at most 4 degenerate bases. Primer sets for amplifying the C_H1_ and C_κ_ domains are listed in Supplementary Table 2 and follow the same naming scheme as below. Primer sets for scFv amplification were designed for maximal coverage of all human immunoglobulin sequences. Consensus nucleotide sequences for leader, variable and constant regions were downloaded from IMGT^37^ for the human heavy, lambda, and kappa genes (excluding pseudogenes and truncated transcripts) and four sets of primers were designed for each gene family. Outside primers (“out” subscript in the primer names; Supplementary Data 1) were designed using Primer3^38,39^ to span the splice junction of the leader sequence (“out_5” primers) or bind within the first 50 bases of the constant domain (“out_3” primers) including, when possible, the V–C splice junction. Inside primers (“in” subscript in the primer names; Supplementary Data 1) were designed by fixing the 5′ end of the primer to the start (“in_5”) or end (“in_3”) of the V coding sequence. FR4-specific primers were also manually extended for increased specificity. The inside PCR primer sets were fused to overhangs to enable linker formation (VH_in_3 and VK/L_in_5), or restriction digestion by NotI or SfiI and barcoded Illumina MiSeq sequencing (Fig. 2a; Supplementary Table 2, Supplementary Table 3 and Supplementary Data 1).

### Validation of native chain pairing of antibodies

Informed consent was obtained from all subjects prior to human blood collection and the process was approved under the Chesapeake IRB (protocol number 2010-001). Primary human B cells were isolated from 80 ml human blood samples using the RoboSep Human B Cell Enrichment Kit (StemCell Technologies). Primary mouse B cells were isolated from splenocytes using the Mouse B cell Isolation Kit (StemCell Technologies) according to the manufacturer’s instructions. Isolated cells were centrifuged at 500 × *g* for 10 min and re-suspended in RPMI1640 (Invitrogen), supplemented with insulin-transferrin-selenium (Invitrogen), 10% fetal bovine serum (Invitrogen), 0.5 μg ml^−1^ megaCD40L (Enzo), 33 ng ml^−1^ IL-21 (produced in-house) and penicillin–streptomycin–glutamine (Invitrogen), and incubated at 37 °C and 5% CO_2_ for 48 h. Mouse and human B cells were combined in a 1:1 ratio and 10,000 cells from this mixture were encapsulated as described below. A parallel “combinatorial” reaction was performed by combining 10,000 cells directly in RT-PCR mix (described below) without encapsulation. RT-PCR and nested PCR conditions were as described below using primer sets designed to amplify and link the C_H1_ and C_κ_ domains (Supplementary Table 2).

### Encapsulation of primary human B cells

Total B cells from healthy donors were isolated and stimulated as described above. For memory B cell isolation, we further used the Human Switched Memory B Cell Isolation Kit (Miltenyi Biotec). Two million cells were washed in PBS (3 min at 700 g) and split into two halves: one million cells were processed for total RNA using the RNEasy RNA isolation kit (Qiagen) according to the manufacturer’s instructions. The remaining one million cells were re-suspended in 250 μl encapsulation buffer: hypo-osmolar electrofusion buffer (Eppendorf cat no 940002001) containing 1:1000 dilution of Anti-Clumping Agent (Invitrogen cat no 01-0057AE) and 16% OptiPrep Density Gradient medium (Sigma cat no D1556). This cell concentration resulted in one cell being encapsulated in every 10 droplets on average. Theoretically, this means that the number of cells encapsulated in a single droplet follows a Poisson distribution:

$$p(k) = \frac{{\lambda ^ke^{ - \lambda }}}{{k!}}\,{\mathrm{, where}}\,{\mathrm{\lambda = 0}}{\mathrm{.1}}$$

From this we calculate the percentage of single-cell droplets out of all non-empty droplets as follows:

$$\frac{{p\left( {k = 1} \right)}}{{1 - p(k = 0)}} = 95.08{\mathrm{\% }}$$

Cells were encapsulated at a 1:1 ratio with 2× RT-PCR master mix. The primers within each set were mixed in equal amounts and optimized concentrations of each set were added to the RT-PCR mix. The 2× RT-PCR master mix was composed of 139 nM VH-out-F, 416 nM VL-out-R, 39 nM VH-in-R, and 13 nM VL-in-F (Supplementary Data 1), 2× One Tube RT-PCR reaction buffer (Roche cat no 11855476001), 4% Titan One Tube RT-PCR enzyme mix (Roche cat no 11855476001), 18.2% Q solution (Qiagen cat no 210212), 0.4 mM dNTP (Invitrogen cat no 18427013), 10 mM DTT (Roche cat no 11855476001), and 120 units RNaseOUT (Invitrogen cat no 10777019).

Encapsulation was performed on a 2-reagent droplet generation fluorophilic chip (Dolomite cat no 3200510) with fluids pumped using an OB1 flow controller (Elveflow cat no MKII). Aqueous liquids of cells and RT-PCR mix were each pumped at 30 mbar while HFE7500 fluorinated oil + 2% w/v 008-fluoro-surfactant (RAN Biotechnologies cat no 008-FLUOROSURFACTANT-HFE7500) was pumped at 67 mbar, with pressures fine-tuned to obtain a 1:1 mix of aqueous fluids. The resulting emulsion was collected in fractions (about 40 μl emulsion per fraction) in PCR strip tubes and overlaid with 40 μl mineral oil. Excess fluorinated oil was removed to maintain an overall volume of about 100 μl.

In parallel, we created a combinatorial library from the purified RNA. In total, 250 ng total RNA was used for RT-PCR using the same master mix as with emulsions, except that the V_H_ and V_L_ sequences were amplified separately and then paired by overlap-extension PCR, as described below.

### Amplification of Scfv containing natively paired V-genes

Encapsulated and combinatorial libraries were created by reverse transcription for 30 min at 50 °C followed by 2 min at 88 °C. This was followed by 45 (emulsion) or 35 (combinatorial) cycles of PCR (88 °C for 10 s, 62 °C for 30 s, 68 °C for 45 s) and a final extension step of 7 min at 68 °C. Excess oil below the droplets was manually removed and the droplets chemically coalesced using an equal volume of Pico-Break 1 (Dolomite cat no 3200228). Amplified DNA was electrophoresed on 2% agarose and the region between 650 bp-1000 bp purified using the QIAquick gel-extraction kit (Qiagen).

Nested PCR amplification consisted of 25% purified RT-PCR product, 100 nM VH-in-F and VL-in-R primer pools (Supplementary Data 1), 1× Hifi Platinum PCR buffer, 0.15 mM dNTP, 1.5 mM MgSO_4_, and 0.6 units Hifi Platinum Taq (Invitrogen, cat no 11304011). Cycling conditions consisted of an initial denaturation step of 2 min at 94 °C followed by 50 cycles of PCR (94 °C for 30 s, 55 °C for 30 s, 68 °C for 60 s) and a final extension step of 10 min at 68 °C. Products were again size-selected as above.

A final scale-up PCR was performed using common forward (Illu_scaleup_F) and barcoded reverse primers (Illu_R_N50X) to enable library construction and Illumina sequencing (Supplementary Table 3). We used the Q5 polymerase (NEB, M0491S) according to manufacturer’s instructions with the following thermocycling program: 98 °C for 2 min, 12–20 cycles of 98 °C for 10 s and 72 °C for 30 s, 72 °C for 2 min.

### Imaging of single encapsulated B cells

B cells were stained using CellTracker Red CMTPX or CellTracker Green CMFDA dyes (Life Technologies) according to the manufacturer’s instructions. Stained cells were re-suspended in PBS and encapsulated using the conditions described above, substituting the RT-PCR mix with PBS. Bulk cell lysis was measured by counting 100,000 cells on the ViCell Cell Viability Analyzer (Beckman Coulter) using Trypan Blue staining and default settings, or by incubating the same number of cells in either 100 μl culture media or RT-PCR buffer and imaging cells in a 96 well U-bottom plate (Corning) at 40x magnification. Cell lysis in droplets was imaged in two ways: (1) stained cells were re-suspended in encapsulation buffer, encapsulated with RT-PCR mix and heated to 50 °C for 5 min; (2) unstained cells were encapsulated with RT-PCR mix containing 2× SYBR-Green (Invitrogen) and heated to 50 °C for 5 min. Droplets were collected in μ-Slide^0.1^ channel slides (Ibidi) and imaged at 200x magnification using the Evos FL Auto Cell Imaging System (Invitrogen).

### Next-generation sequencing and bioinformatic analysis

Each barcoded library was size-selected to 850 bp by 6% polyacrylamide gel electrophoresis, quantified using the Agilent TapeStation, combined in equal amounts, and subjected to 2 × 300 bp MiSeq sequencing using a custom priming approach (SeqMatic). We performed two forward 300 bp reads with the R1 and R2 primers (Supplementary Table 3) by modifying the instrument sample sheet to replace the index1 read with our custom primer and generate the V_L_ sequence. The index2 read used the standard Illumina P5 primer. Following demultiplexing, raw Fastq reads were quality-filtered using FastQC, paired by the Illumina Fastq ID, and aligned to IMGT V and J genes using IgBLAST^19^ to annotate germline families and delineate CDR3 regions. Subsequently, CDRH3 and CDRL3 sequences were concatenated and clustered where the amino acid identity was greater than 88%. Unique CDRH3 and CDRL3 sequences were counted and the numbers of unique V_L_ sequences pairing with each unique V_H_ were used as a measure of pairing efficiency.

For CDRH3 sequences paired with multiple CDRL3, the top-pair weight is determined as the ratio of counts between the most abundant CDRL3 and all CDRL3 sequences^13^. Briefly, for each unique CDRH3, we counted associated unique CDRL3 sequences. If a CDRH3 has *m* CDRL3 pairs and the count of these pairs are *c*_i_ (1 ≤ *i* ≤ *m*), then the top-pair count of this CDRH3 is defined as $\max \left( {c_{\rm {i}}} \right)$ and top-pair weight is $w = \frac{{{\mathrm{max}}(c_{\rm i})}}{{{\rm sum}(c_{\rm i})}}$. The calculation is performed for all the unique CDRH3, excluding singletons, since such sequences would all have a top-pair weight of 1. Ideally if one CDRH3 is uniquely paired with one CDRL3, the top-pair weight should be 1 (i.e., $\max \left( {c_{\rm i}} \right) = {\rm sum}(c_{\rm i})$).

Statistical comparison of top-pair distributions were performed using the Mann–Whitney non-parametric test (one-sided, unpaired) using the R stats function “wilcox.test”.

### Phage-display library construction and enrichment for anti-hemagglutinin antibodies

Amplicon libraries were subcloned into pCANTAB6 using Not1 and Sfi1 restriction enzymes (New England Biolabs) and phage-display libraries were generated as previously described^17^. A total of 96 colonies from each of the four libraries were cultured to mid-log phase and infected with M13-K07 (Invitrogen) to initiate overnight monoclonal phage production. Antibody display was determined by ELISA: 1 μg ml^−1^ anti-myc antibody (Invitrogen) was immobilized overnight on 96 well MAXISORP plates (Nunc) and blocked for 2 h with 3% BSA (Sigma) and 0.05% Tween-20 (BDH). Following washing with PBST (PBS pH 7.2 (Invitrogen) + 0.05% Tween-20), diluted phage supernatant was bound and detected using an anti-M13-HRP antibody (1:5000, GE Healthcare, cat no 27942101) and visualized with TMB (KPL, cat no 52-00-01).

Recombinant hemagglutinin proteins were expressed and purified as previously described^40^. HA proteins used as follows; H1 CA/09, A/California/07/2009 H1N1; H1 SD/07, A/South Dakota/06/2007 H1N1; H2 MO/06, A/Swine/Missouri/2006 H2N3; H5 VN/04, A/Vietnam/1194/2004 H5N1; H6 HK/97, A/teal/Hong Kong/W312/97 H6N1; H9 HK/97, A/chicken/Hong Kong/G9/97 H9N2; H3 PE/09, A/Perth/16/2009 H3N2; H7 NL/03, A/Netherlands/219/2003 H7N7; B FL/06, B/Florida/04/2006 Yamagata lineage; B BR/08, B/Brisbane/60/2008 Victoria lineage.

Phage-display libraries were enriched over two rounds of panning as previously described^17^. The first round used 75 nM biotinylated hemagglutinin H1 (A/California/07/2009 H1N1), while the second round was panned for cross-reactive clones using 75 nM hemagglutinin H5 (A/Vietnam/1203/2004 H5N1). Amplified phage outputs were profiled by polyclonal ELISA as above, using immobilized NeutrAvidin (Thermo Fisher Scientific) to capture specific biotinylated antigen prior to incubation with phage.

NGS libraries from panning outputs were prepared by amplifying DNA purified from the panning outputs with the phagemid-specific primers pCANTAB6-F and pCANTAB6-R (Supplementary Table 3) with Q5 polymerase (NEB) according to manufacturer’s instructions with the following thermocycling program: 98 °C for 30 s, three cycles of 98 °C for 10 s, 65 °C for 20 s and 72 °C for 60 s, and a final 72 °C extension for 2 min. This was followed by Illumina barcoding PCR using the same primers and conditions as above (Illu_scaleup_F + Illu_R_N50X—Supplementary Table 3) but using only 10 cycles of amplification.

Enriched libraries were subcloned into an scFv-Fc expression vector^21^ using Not1 and Sfi1 restriction enzymes (New England Biolabs) and transformed into chemically competent Top10 cells (Invitrogen). Single clones were grown overnight in LB containing 100 μg ml^−1^ carbenicillin (Invitrogen) and 2% Glucose (TekNova) before being diluted 1:500 in reconstituted MagicMedia (Invitrogen) containing 100 μg ml^−1^ carbenicillin (Invitrogen). Cells were induced for 72 h at 25 °C and pelleted by centrifugation. Diluted supernatants were used to determine antigen reactivity by ELISA as described above, using an anti-Fc-gamma-HRP secondary antibody (Jackson ImmunoResearch). Following Sanger sequencing of identified hits, unique clones were expressed in HEK-293 cells for 6 days and supernatants were used to determine EC_50_ values by ELISA.

### Measurement of binding affinity and epitope binning by biolayer inferometry

All binding steps were performed in PBS pH 7.2 containing 3% BSA (Sigma) and 0.05% Tween-20 (BDH). ScFv-Fc antibodies at 10 μg ml^−1^ were immobilized on Protein A biosensors (Forte Bio cat no 18-5010) for 300 s before an equilibration step for 180 s. Bound antibodies were associated with a minimum of 5 concentrations of biotinylated hemagglutinin H1 (A/California/07/2009 H1N1) or hemagglutinin H5 (A/Vietnam/1203/2004 H5N1) for 300 s, then dissociated in buffer for 600 s or 2000 s for high-affinity antibodies. The affinity of the 0084-B14 antibody was assayed as above, but using Anti-human Fc biosensors (Forte Bio cat no 18-5060). Positive binding was determined where the background-corrected delta value was greater than 0.03 nm. For *K*_D_ determination, curves were fit to a standard 1:1 binding model using analysis software by Forte Bio. Only curves that showed good fits to this model (*R*^2^ > 0.96) were used in affinity calculations.

Competition assays were performed by immobilizing biotinylated antibody to streptavidin biosensors (Forte Bio cat no 18-5019) at 10 μg ml^−1^ for 1200 s before equilibrating the sensors in buffer containing 250 μM d-biotin (Amresco) for 180 s. Association was performed with 1 μM hemagglutinin H1 (A/California/07/2009 H1N1) or buffer for 500 s before associating with competing IgG for 500 s.

### Viruses and microneutralization assay

Wild-type influenza strains were obtained from the Centers for Disease Control and Prevention or purchased from the American Tissue Culture Collection. All viruses were propagated in embryonated chicken eggs, and virus titers were determined by mean 50% tissue culture infective dose (TCID50) per milliliter. The microneutralization assay was performed as described previously^40^. Briefly, 60 TCID50 of virus was added to each well in three-fold serial dilutions of antibody (starting from 1 mg ml^−1^) in a 384-well plate in complete minimal essential medium containing 0.75 μg ml^−1^ Trypsin (Worthington) in duplicate wells. After 1 h incubation at 33 °C 5% CO_2_, 2 × 10^4^ MDCK cells per well were added to the plate. Plates were incubated at 33 °C 5% CO_2_ incubator for approximately 40 h, and the neuraminidase (NA) activity was measured by adding a fluorescently labeled substrate, methylumbelliferyl-N-acetyl neuraminic acid (Sigma) to each well and incubated at 37 °C for 1 h. Virus replication represented by NA activity was quantified by reading fluorescence using the following settings: excitation 355 nm, emission 460 nm; 10 flashes per well.

### Data availability

The NGS data generated during the current study are available at the SRA archive (Study accession: SRP104286). Source code of the analysis methods used in this study are available for use under the GPL v.3 license at GitHub: https://github.com/Jincheng2009/igseqanalysis.

## Electronic supplementary material

Supplementary Information

Description of Additional Supplementary Files

Supplementary Data 1

Supplementary Data 2
