# Supplementary material for: Recombinant human B cell repertoires enable screening for rare, specific, and natively paired antibodies
Source: Commun Biol. 2018 Jan 22;1:5. doi: 10.1038/s42003-017-0006-2 (PMC6123710; doi:10.1038/s42003-017-0006-2)
Supplement: Supplementary file 1 — Supplementary Information [file 42003_2017_6_MOESM1_ESM.pdf]

<u>**Supplementary Information**</u>

SUPPLEMENTARY FIGURES

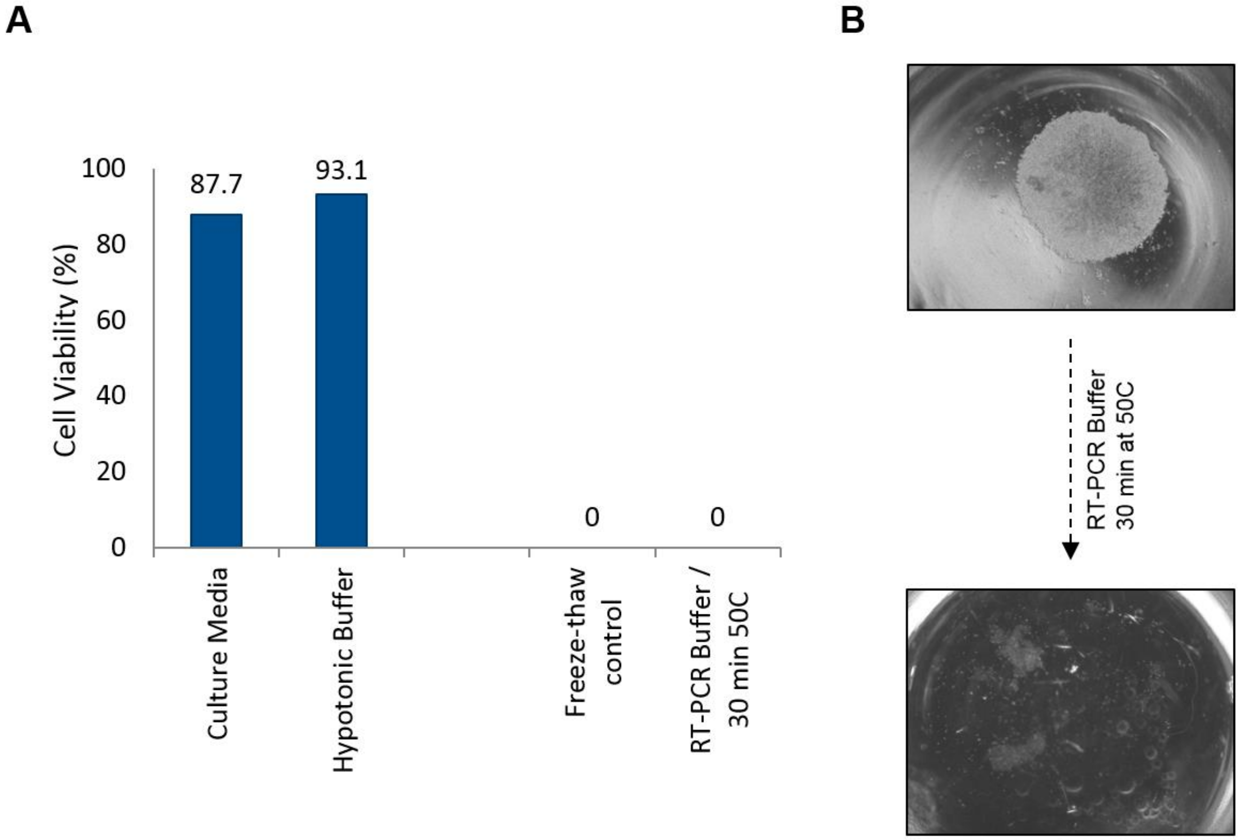

Supplementary Figure 1 Primary B cells are efficiently lysed during conditions of reverse transcription. (A) Cell viability measurements were performed using the ViCell viability analyzer on cells incubated for 30 minutes in either culture medium, encapsulation buffer ("Hypotonic Buffer") and RT-PCR buffer. Viability after one freeze-thaw cycle was also measured for comparison. (B) Visual examination of cell lysis. Cells were stained with Trypan Blue and imaged before and after incubation with RT-PCR buffer for 30 minutes at 50°C.

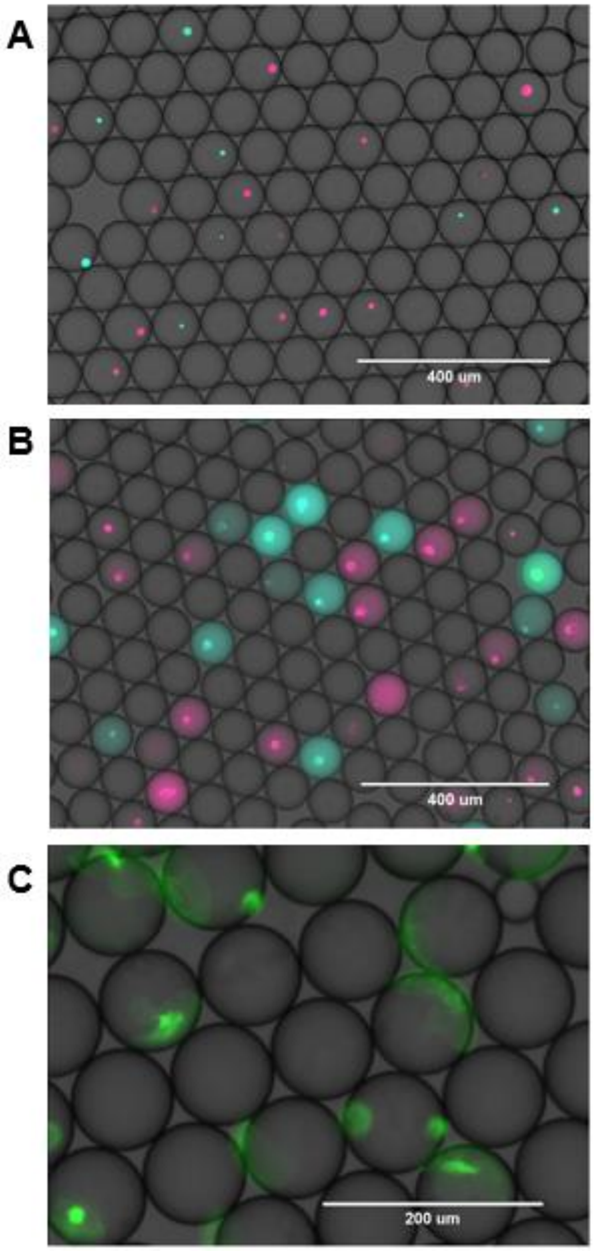

Supplementary Figure 2 Single cells are efficiently lysed within droplets with RT-PCR reagents. IM9 multiple myeloma cells were stained with CellTracker Red or Green (pseudocolored magenta and cyan, respectively) and encapsulated with PBS buffer (A) or RT-PCR buffer (B) shortly before imaging. (C) Single cells were encapsulated with RT-PCR reagents and SYBR-Green dye to visualize the release of double-stranded nuclear material upon cell lysis.

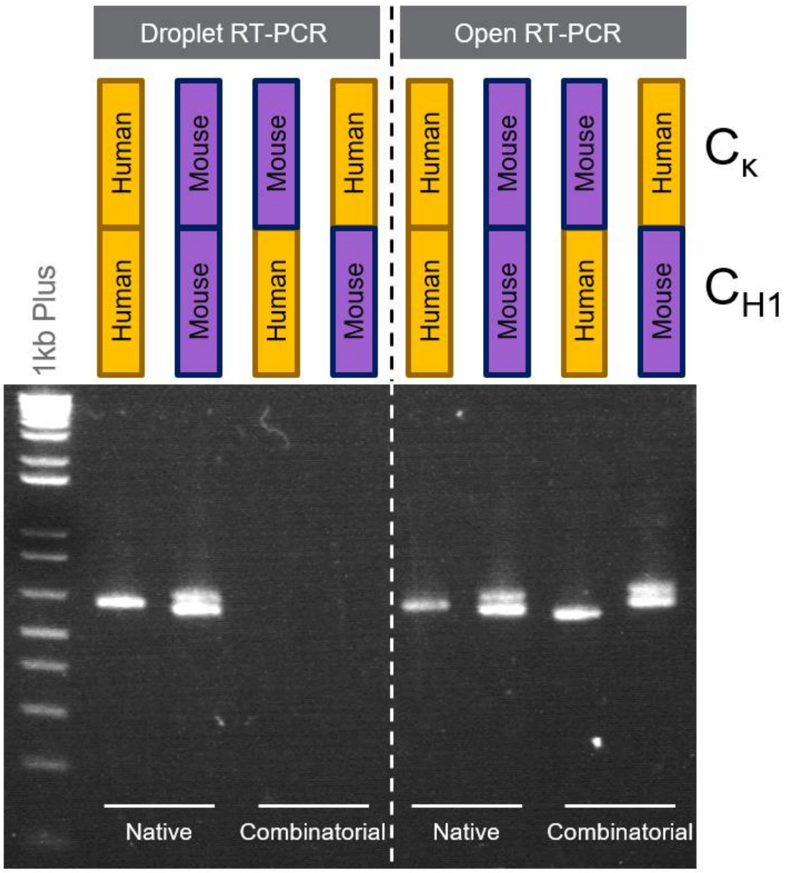

Supplementary Figure 3 Validation of native chain pairing during droplet RT-PCR. Primary human and mouse B cells were mixed and their C_{H1}-C_K domains linked in either encapsulated ("droplet") or pooled ("open") RT-PCR. The resulting amplicons were purified and chain pairing was determined using specific nested primer combinations. Correctly-paired species was only obtained using encapsulation, whereas the scambled format generated all possibile combinations in relatively equal amounts.

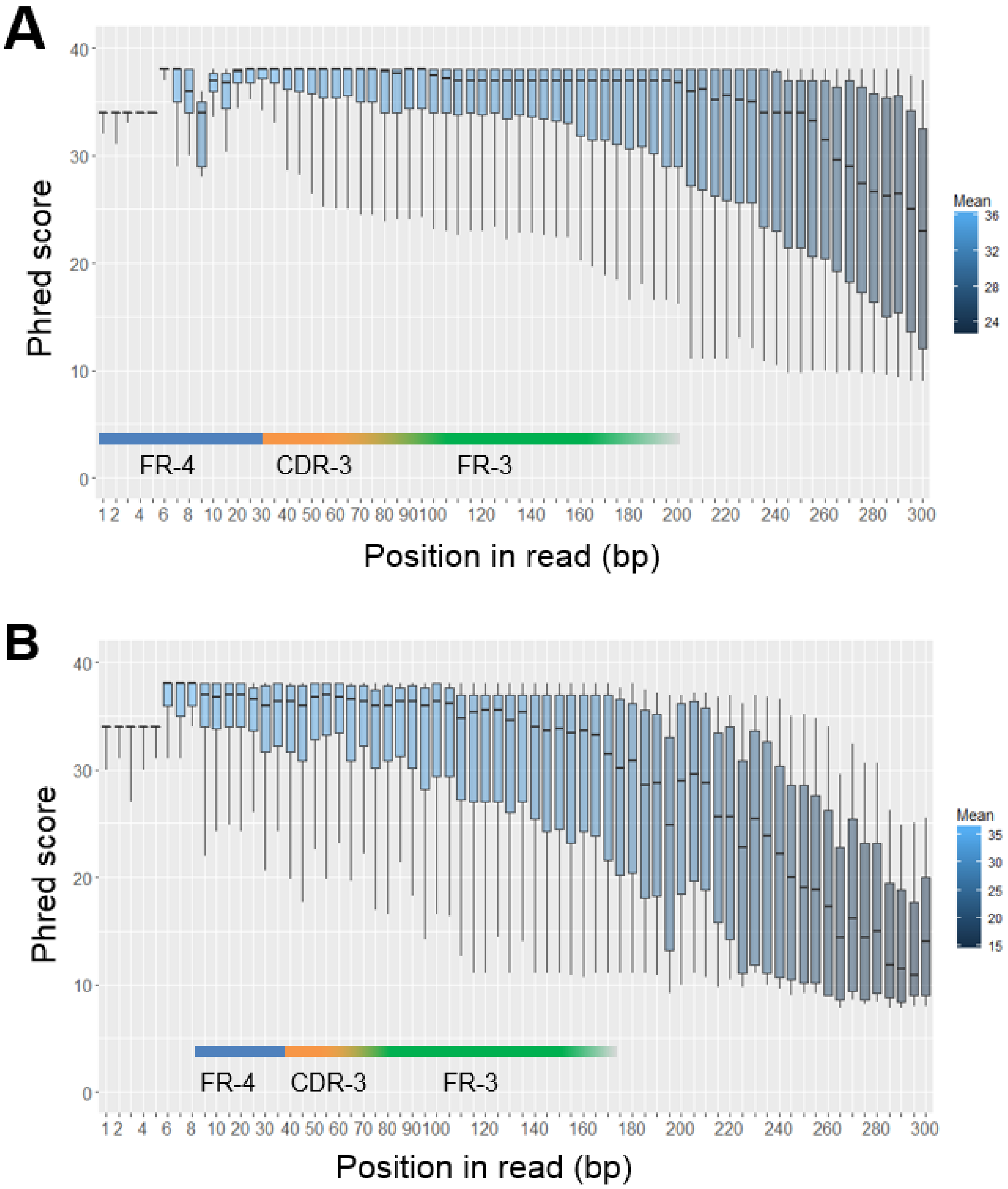

Supplementary Figure 4 Next-generation sequencing quality of paired V_H and V_L reads. Representative sequencing Phred quality scores of (A) V_H and (B) V_L sequences. Framework 4 (FR-4), CDR-3 and Framework 3 (FR-3) regions are highlighted in blue, orange and green, respectively. The observed length diversities in CDR-3 (CDR-H3: 23-74nt; CDR-L3: 14-42nt – further described in Supplementary Figures 4a and 5a) are depicted as gradients.

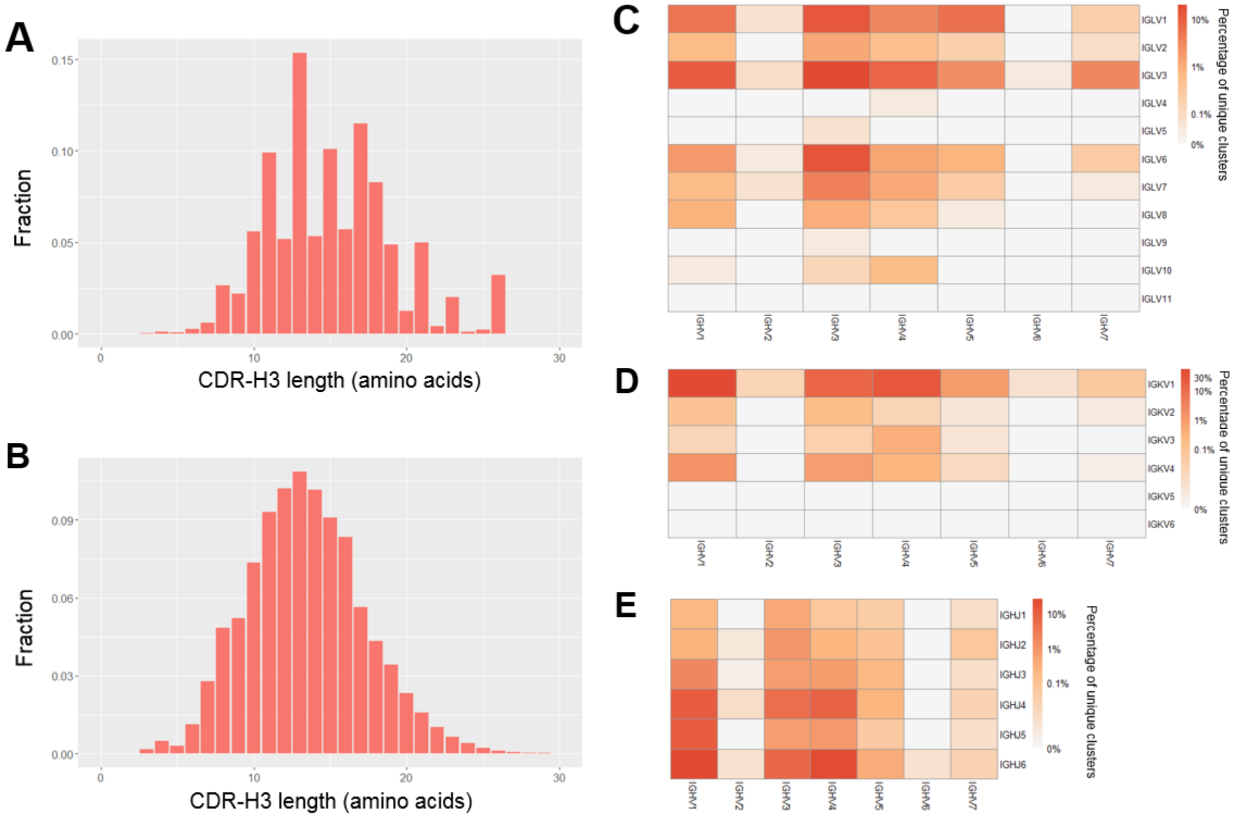

Supplementary Figure 5 Analysis of heavy chain CDR length diversity and V gene germline usage. Annotations of next-generation sequencing data were performed using IgBLAST to delineate CDR3 boundaries and relative distributions of each length are shown for CDR-H3 from (A) emulsion and (B) combinatorial libraries. V-gene germline families were also identified using IgBLAST and the pairwise relative abundance among unique clusters is plotted as a heat map for (C) V_H-V_λ, (D)V_H-V_κ and (E) V_H- J_H.

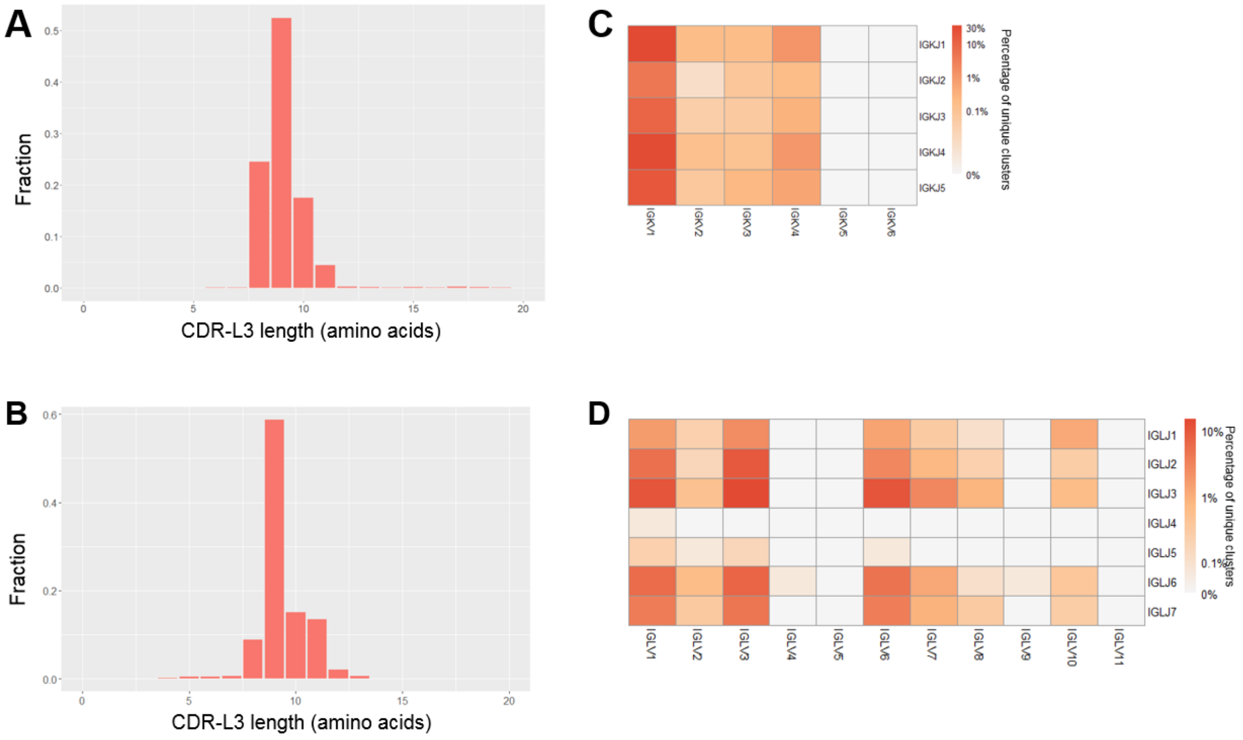

Supplementary Figure 6 Analysis of light chain CDR length diversity and V gene germline usage. Annotations of next-generation sequencing data were performed using IgBLAST to delineate CDR3 boundaries and relative distributions of each length are shown for CDR-L3 from (A) emulsion and (B) combinatorial libraries. V-gene germline families were also identified using IgBLAST and the pairwise relative abundance among unique clusters is plotted as a heat map for (C) V_κ-J_κ and (D) V_λ-J_λ.

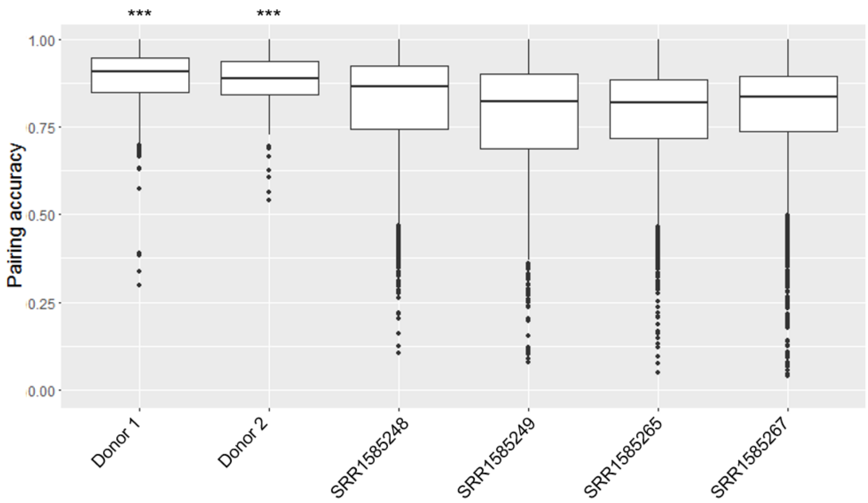

Supplementary Figure 7 V_H-V_L pairing accuracy from top-pair analysis comparing antibody libraries generated within droplets from this study (lanes 1-2) and a reference study[13] (lanes 3-6). Cell number and sequencing depth are comparable between samples. *** p<0.001 between Donor 1 or Donor 2 and any of the other libraries using the non-parametric Mann-Whitney test (one-sided, unpaired).

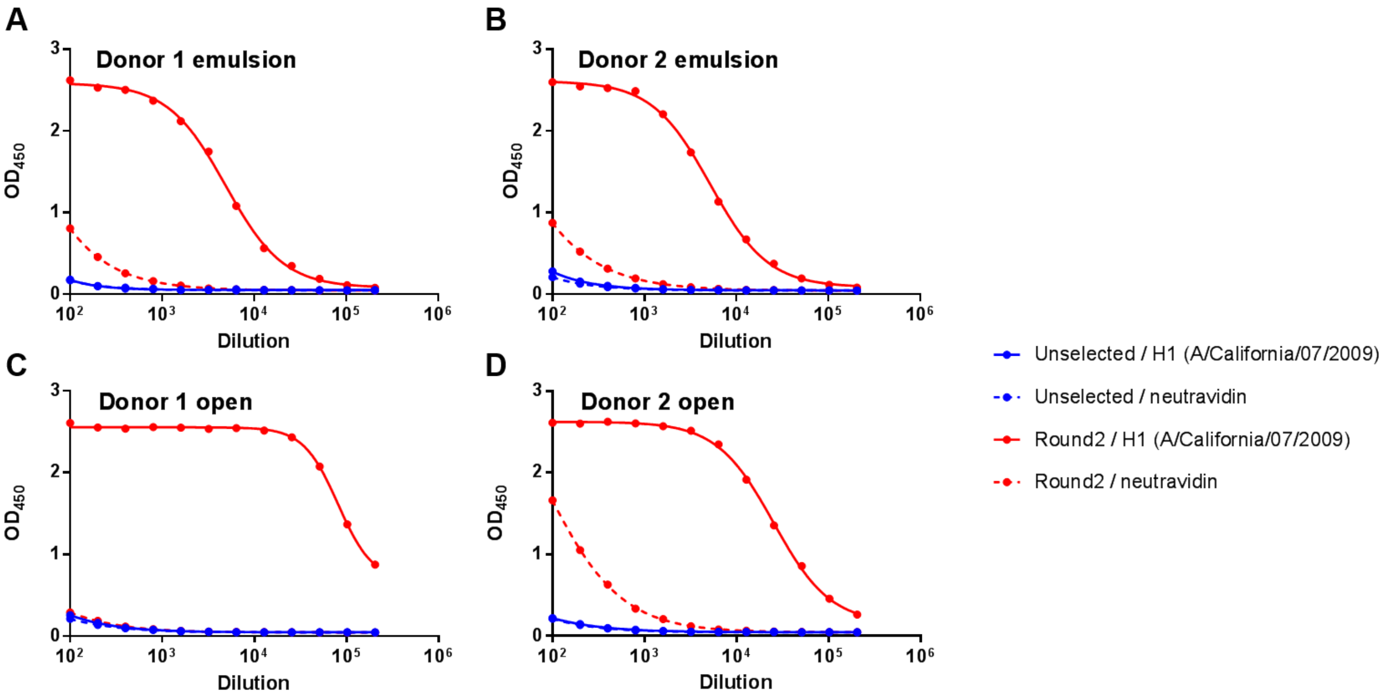

Supplementary Figure 8 Polyclonal phage ELISA showing enrichment after two rounds of selection on hemagglutinin. Unselected library is shown in blue, whereas the round 2 selected library is shown in red. Binding of diluted phage was measured against hemagglutinin H1 ((A/California/07/2009 H1N1 - solid lines) or an irrelevant protein control (dashed lines) for libraries generated from emulsified total B cells (A), emulsified memory B cells (B), combinatorial total B cells (C) or combinatorial memory B cells (D).

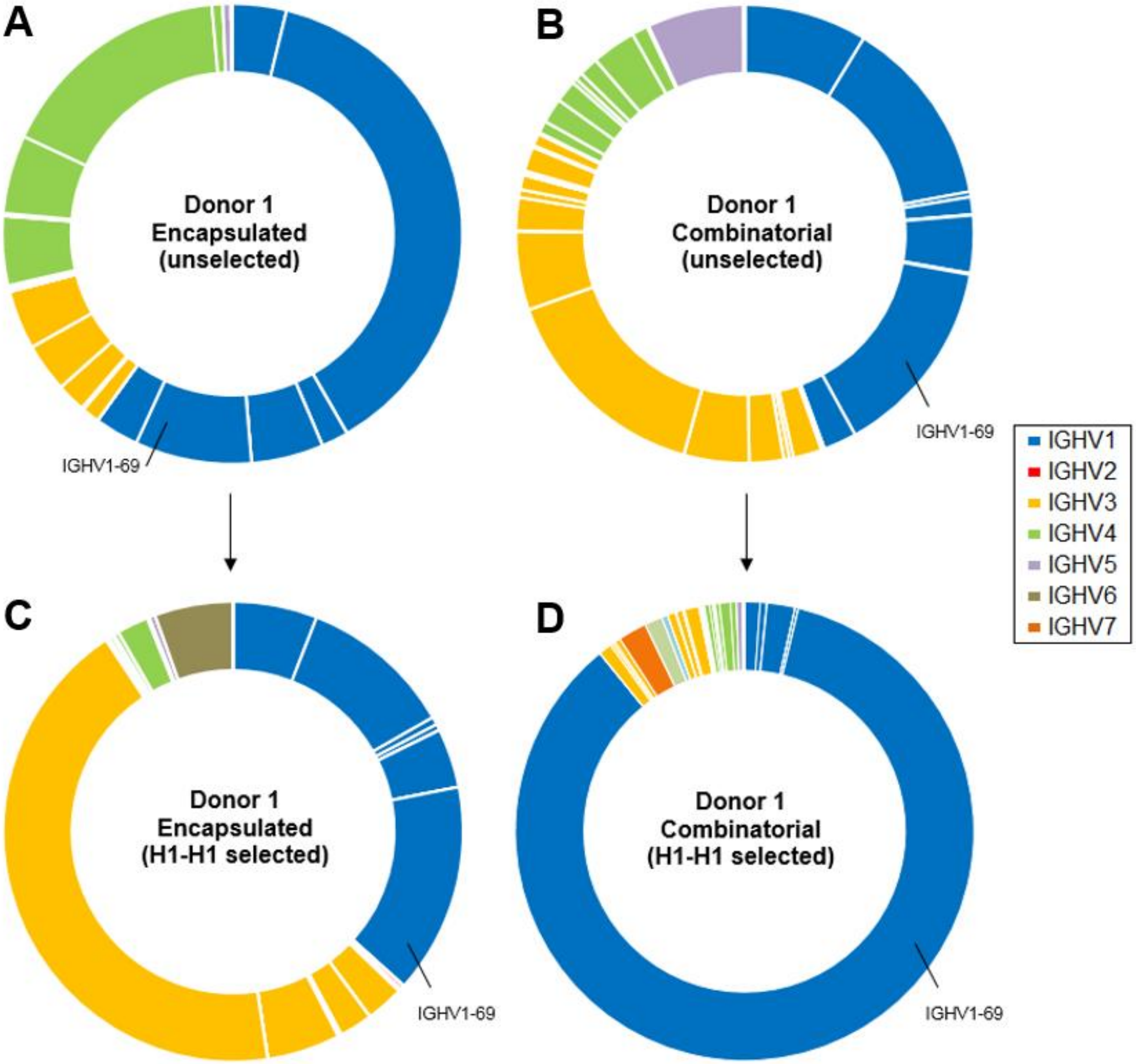

Supplementary Figure 9 VH germline distribution before and after 2 rounds of panning on hemagglutinin H1 (A/California/07/2009 H1N1). Libraries from the same donor were generated in either encapsulated (A, C) or combinatorial (B, D) formats and relative abundance of each V_H germline family determined by next-generation sequencing. The relative abundance of IGHV1-69, a germline family that encodes antibodies known to contact group 1 hemagglutinin through heavy chain interactions alone, is indicated within each plot.

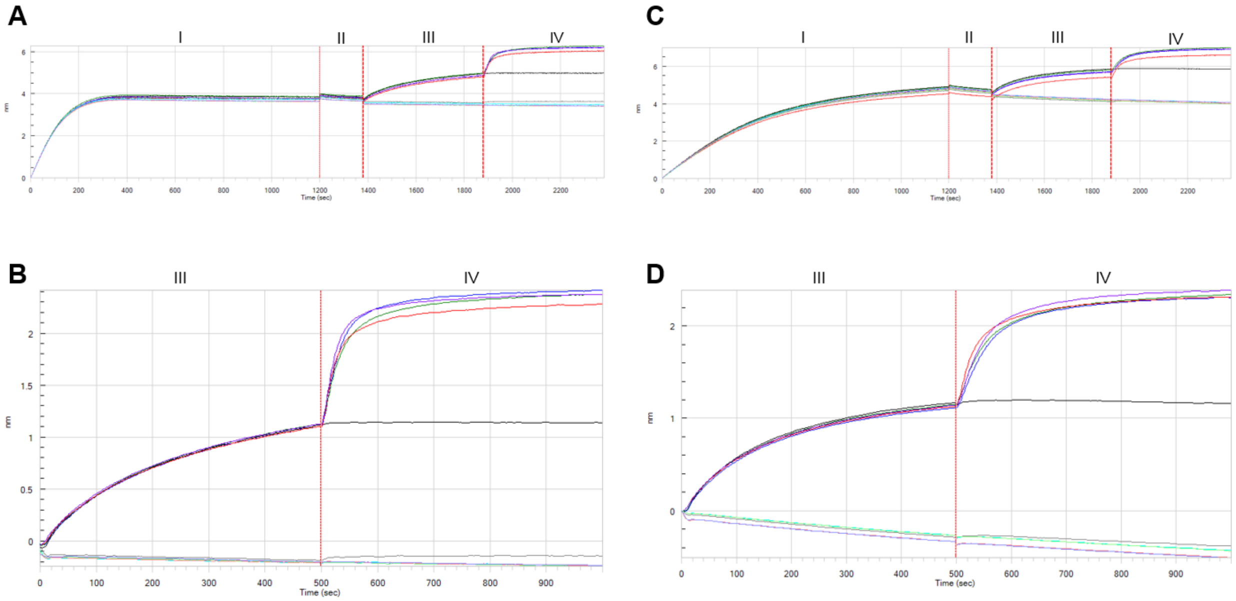

Supplementary Figure 10 Broadly cross-reactive anti-hemagglutinin antibodies discovered in the current study do not compete with known cross-reactive antibodies. Biolayer inferometry was used for competition experiments by immobilizing biotinylated 0089AY-D17 (A,B) or 0084GM-D06 (C,D) on Streptavidin biosensors (step I), equilibrating the sensors in buffer and free biotin (step II), binding hemagglutinin H1 (A/California/07/2009 H1N1 – step III) and measuring concurrent binding with FI6v3 (blue), MEDI8852 (green), 1N23 (red), CR9114 (magenta) or unlabeled antibody (black). The other traces (dimmed colors) are for competition performed in the absence of antigen. Figures B and D depict magnified views of figures A and C, respectively.

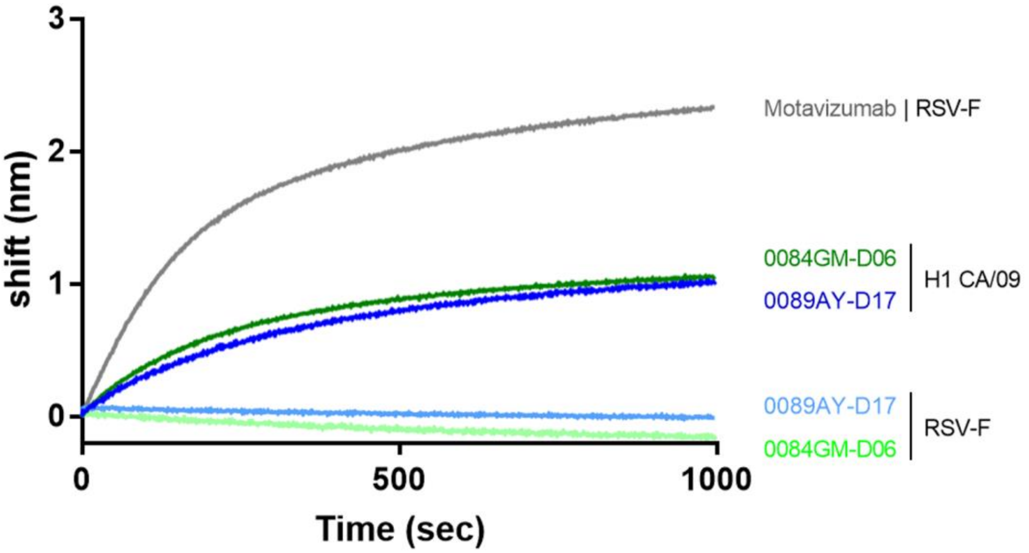

Supplementary Figure 11 Broadly cross-reactive antibodies do not bind to Foldon-His domains. Biolayer inferometry was used to measure binding of 0089AY-D17 (blue) or 0084GM-D06 (green) to either hemagglutinin H1-foldon-his (A/California/07/2009 H1N1 – darker colors) or an irrelevant protein fused to the same Foldon-His domains (RSV-F – lighter colors). The integrity of the fused RSV-F protein was confirmed using the anti-RSV-F antibody Motavizumab (grey).

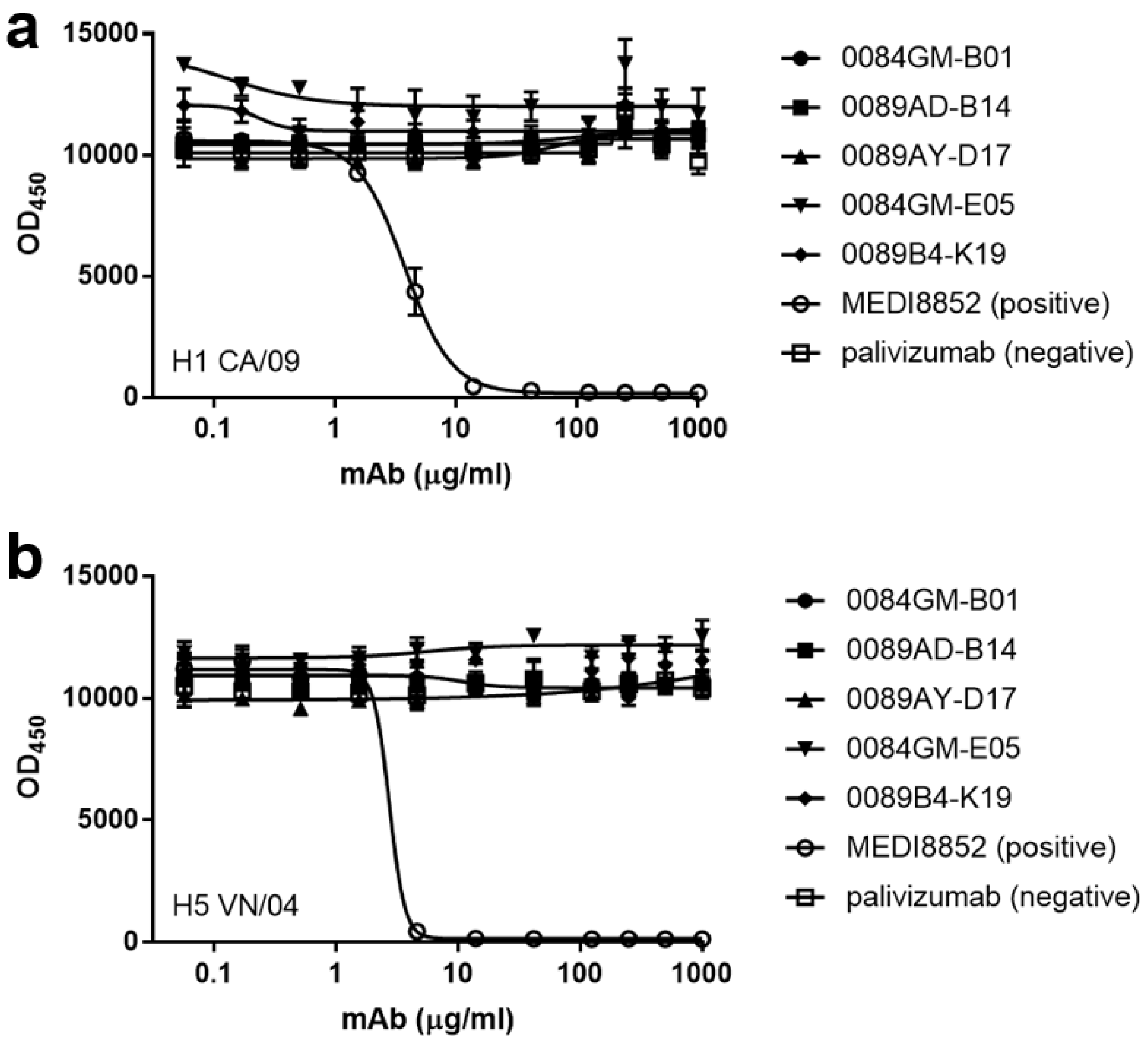

Supplementary Figure 12 H1 and H5 cross-reactive antibodies do not show neutralization activity. Micro-neutralization assays were performed using IgG-converted antibodies against the seasonal H1N1 A/California/07/2009 virus (a) or non-seasonal H5N1 A/Vietnam/1203/2004 virus (b). The broadly neutralizing anti-influenza virus antibody MEDI8852 (open circles) and anti-respiratory syncytial virus antibody palivizumab (open squares) are shown as positive and negative controls, respectively.

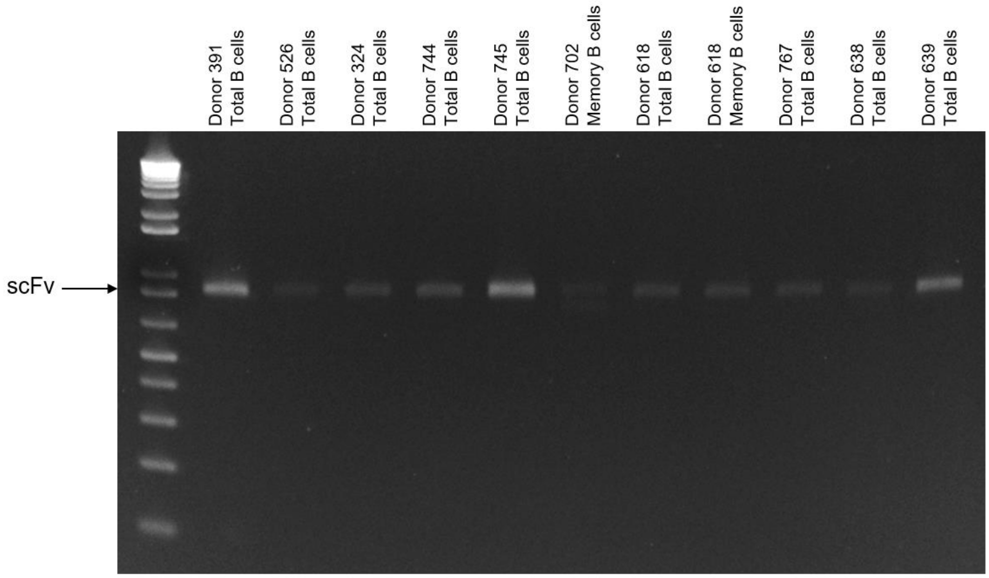

Supplementary Figure 13 Natively-paired scFv amplicon libraries generated from eleven separate encapsulations comprising different donors and B cell subsets.

SUPPLEMENTARY TABLES

Supplementary Table 1 Comparison of twelve One-Step RT-PCR kits for compatibility with droplet RT-PCR. Each kit was used to generate droplets with IM-9 myeloma cells and primers to amplify GAPDH and stability of the droplets was determined at 23°C and 88°C. Of the kits stable at 88C, the presence of human GAPDH amplicon was determined by agarose electrophoresis.

	**Vendor**	**Product**	**Stable at 23°C**	**Stable at 88°C**	**Emulsion RT-PCR product**
1	NEB	OneTaq® One-Step RT-PCR Kit	No	No	nd
2	Invitrogen	SuperScript® One-Step RT-PCR System with Platinum® Taq DNA polymerase	No	No	nd
3	Invitrogen	SuperScript III® HiFi One-Step RT-PCR System with Platinum® Taq DNA polymerase	No	No	nd
4	AffyMetrix	One-Step RT-PCR Kit	No	No	nd
5	Agilent	AffinityScript One-Step RT-PCR Kit	No	No	nd
6	Qiagen	OneStep RT-PCR Kit	No	No	nd
7	Thermo	Verso 1-Step RT-PCR Kit with ThermoPrime Taq	Yes	No	nd
8	Takara	Titanium® One-Step RT-PCR Kit	Yes	No	nd
9	Takara	(AMV) One-Step RNA PCR Kit	Yes	No	nd
10	Roche	Transcriptor	Yes	Yes	No
11	Roche	Titan	Yes	Yes	Yes

Supplementary Table 2 Primers used to amplify and pair human and mouse C_{H1}-C_K domains. Regions of the primer that specifically bind the target gene are shown in uppercase whereas overhangs are shown in lowercase.

Primer Name	Primer Sequence
Hs-CH1-out-5	AAGGGCCCATCGGTCTTC
Hs-CH1-in-5	CACCCTCCTCCAAGAGCAC
Hs-CH1-in-3	gagccacctccgccgctaccgccgcctccagaTCTTGTCCACCTTGGTGTTG
Hs-CK-in-5	agcggcggaggtggctcaggcggtggcggaagtGTGGCTGCACCATCTGTCT
Hs-CK-out-3	TCCCCTGTTGAAGCTCTTTG
Hs-CK-in-3	CTGTTGAAGCTCTTTGTGACG
Mm-CH1-out-5	CGACACCCCCATCTGTCTAT
Mm-CH1-in-5	CCCCCATCTGTCTATCCACT
Mm-CH1-in-3	gagccacctccgccgctaccgccgcctccagaCAATTTTCTTGTCCACCTTGG
Mm-CK-in-5	agcggcggaggtggctcaggcggtggcggaagtCTGTATCCATCTTCCCACCA
Mm-CK-out-3	ACTCATTCCTGTTGAAGCTCTTG
Mm-CK-in-3	GGGTGAAGTTGATGTCTTGTGA

Supplementary Table 3 Primers used in library amplification for phage-display generation or next generation sequencing profiling. Regions of the primer that specifically bind the target gene are shown in uppercase whereas overhangs are shown in lowercase. Specific index sequences are underlined.

Primer Name	Primer Sequence
Illu_R_N505	aatgatacggcgaccaccgagatctacacgtaaggagGATTACGCCAAGCTTTGGAGCC
Illu_R_N506	aatgatacggcgaccaccgagatctacacactgcataGATTACGCCAAGCTTTGGAGCC
Illu_R_N507	aatgatacggcgaccaccgagatctacacaaggagtaGATTACGCCAAGCTTTGGAGCC
Illu_R_N508	aatgatacggcgaccaccgagatctacacctaagcctGATTACGCCAAGCTTTGGAGCC
R1	GATTACGCCAAGCTTTGGAGCC
R2	CGCTACCGCCGCCTCCAGA
Illu_F_scaleup	CAAGCAGAAGACGGCATACGAGAT
IM9-CDRH3-Fwd	GTACAAAAAGAAGGGGGGTGACAGACATTGACCCTTTTG
pCANTAB6-F	cagaagacggcatacgagatggcccagccggccatggccttccttTCTATGCGGCCCAG
pCANTAB6-R	gattacgccaagctttggagccgcggccgcCGTGATGGTGATGATGATGTGC

Supplementary Table 4 Analysis of unique sequences from next-generation sequencing data.

Library	Donor 1 droplet	Donor 1 combinatorial	Donor 2 droplet	Donor 2 combinatorial
**Mapped Reads (V_H)**	3,311,883	1,867,114	2,841,606	3,534,010
**Mapped Reads (V_L)**	2,397,908	898,120	1,981,098	2,553,130
**Mapped Reads (V_H-V_L)**	2,649,093	981,684	2,171,741	2,645,525
**Unique CDR-H3:CDR-L3**	274,979	931,779	206,225	2,171,198
**CDR-H3 clusters**	152,032	445,249	101,488	376,885
**CDR-L3 clusters**	45,444	160,243	38,326	176,520
**CDR-H3:CDR-L3 clusters**	121,845	766,146	90,173	1,783,269
**V_L:V_H ratio (median)**	2	9	2	5

Supplementary Table 5 Statistical comparison of V_H-V_L pairing accuracy from top-pair analysis using the non-parametric Mann-Whitney test (one-sided, unpaired). Antibody libraries generated within droplets are compared between this study (Donor 1 and Donor 2) and a reference study[13] (datasets SRR1585248, SRR1585249, SRR1585265 and SRR1585267). Cell number and sequencing depth are comparable between samples.

p-value	**SRR1585267**	**SRR1585265**	**SRR1585248**	**SRR1585249**
**Donor 1**	9.26E-54	7.03E-61	2.68E-21	3.26E-43
**Donor 2**	1.83E-43	7.94E-53	7.17E-14	2.06E-36

Supplementary Table 6 Phage-display library characterization

Phage Library	B cell Source	Library Size	Clones Displaying scFv (%)
Donor 1 Emulsion	Total B Cells	$3.0x10^8$	98.9
Donor 1 Combinatorial	Total B Cells	$3.0x10^8$	94.7
Donor 2 Emulsion	Memory B Cells	$3.5x10^8$	90.5
Donor 2 Combinatorial	Memory B Cells	$1.0x10^8$	95.7
