# Supplementary material for: Recombinant human B cell repertoires enable screening for rare, specific, and natively paired antibodies
Source: Commun Biol. 2018 Jan 22;1:5. doi: 10.1038/s42003-017-0006-2 (PMC6123710; doi:10.1038/s42003-017-0006-2)
Supplement: Supplementary file 2 — Description of Additional Supplementary Files [file 42003_2017_6_MOESM2_ESM.pdf]

File Name: Supplementary Data 1
Description: Primers used for V/J gene amplification. Regions of the primer that specifically bind the target gene are shown in uppercase whereas overhangs are shown in lowercase. Target V/J genes are listed, with the leader sequence for each gene designated by the suffix "_ldr".

File Name: Supplementary Data 2
Description: Isolated antibody CDR3 lengths, germline usage and scFv nucleotide sequences.
